# What are the goals of sustainable management for problems surrounding dialysis?

**DOI:** 10.1186/s41100-022-00456-x

**Published:** 2023-01-16

**Authors:** Ken Tsuchiya

**Affiliations:** Department of Blood Purification, Tokyo Women’s Medical University, Tokyo, 162-8666 Japan

The 67th Annual Meeting of the Japanese Society for Dialysis Therapy (JSDT) was held in Yokohama, Japan, from July 1 to 3, 2022, with the following theme: “In Pursuit of Sustainable Development Goals (SDGs) for Dialysis Therapy” [1]. JSDT was founded in 1968 as an academic society for research on dialysis and has approximately 18,000 members [2]. The society has worked to establish and advance hemodialysis as a mainstay of renal replacement therapy (RRT). At present (as of December 2020) [3], there are 347,671 patients on chronic dialysis in Japan and hemodialysis is the most common modality. This is the underlying cause of the extreme bias toward hemodialysis over peritoneal dialysis and renal transplantation, and is both a medical and social problem. Therefore, the JSDT has expanded its scope beyond its previous focus on hemodialysis alone into the selection of RRT modalities. This is not only a result of the JSDT re-envisioning its own identity but also a response to social needs.

Around 2002, the term “chronic kidney disease” (CKD) was proposed as a general term for kidney-related diseases, including end-stage kidney disease (ESKD), which requires RRT [4]. Important issues in CKD therapy include not only the diagnosis and treatment of the causative disease, or investigation of the nature of the process that originally caused kidney failure leading to ESKD, but also assessment and management of systemic complications (such as cardiorenal syndrome) that may result from pathophysiological loss of renal function. Other important issues are the RRT to be performed for ESKD, the selection of the modality, and the medical cost burden of continuing RRT. What was formerly known as “kidney disease” has undergone a major transformation into CKD, a concept that encompasses a wide range of problems.

This meeting’s theme, “In Pursuit of SDGs for Dialysis Therapy,” refers to the work of identifying and addressing issues surrounding CKD and RRT. The main issues are listed below:

## Coronavirus disease 2019 (COVID-19) pandemic

An outbreak of respiratory illness that emerged from Wuhan, Hubei Province, China, in December 2019 was named novel coronavirus disease 2019 (COVID-19) and was classified as a pandemic by the World Health Organization (WHO). In Japan, COVID-19 was first detected on January 15, 2020 in a patient with pneumonia who had with a history of travel to Wuhan, and a positive COVID-19 result was first reported in a dialysis patient on March 1, 2020. CKD itself is considered the greatest risk factor for developing severe COVID-19 pneumonia: the mortality rate was initially reported to be nearly 20% in dialysis patients (vs. 1.3% in the general population), and increases with age [5]. In response, the Japanese government has taken proactive measures such as hospitalization, prioritized vaccination, and outpatient antibody therapy for dialysis patients, which has reduced the mortality rate.

COVID-19 is just one example. In fact, hemodialysis, especially in-clinic hemodialysis as is done in Japan, has a number of aspects that increase the risk for infectious diseases in general, including patient drop-off and pick-up, prolonged treatment in the same room, and frequent contact with staff. Beyond infection, the procedure of hemodialysis itself has been noted to have another major downside: it requires large amounts of water and a source of electricity, which makes it extremely vulnerable to external factors such as earthquakes, typhoons, and other disasters, as well as large-scale power outages. In Japan, where the majority of patients depend on hemodialysis for RRT, COVID-19 brought attention to the importance of therapy selection and the need for diversification.

## Aging of the patient population

It has long been noted that the age of patients on maintenance dialysis has been increasing, along with patient age at initiation of dialysis. At the end of 2020, the mean age of dialysis patients was 69.40 years overall, 68.60 years for men, and 70.97 years for women, and the mean age of patients starting dialysis was 70.88 years overall, 70.19 years for men, and 72.48 years for women [6]. Aging can cause physical and mental function to decline in patients over the course of CKD treatment from conservative therapy to dialysis, and poses a risk of comorbidities such as frailty and sarcopenia [7]. The importance of renal rehabilitation has been noted [8], but the COVID-19 pandemic has made rehabilitation impossible to implement properly. It is also questionable whether conventional guidelines for conditions such as anemia and diabetes in various populations of dialysis patients are directly applicable to elderly adults, especially those aged 75 years or older.

## Forgoing dialysis and selection of conservative kidney management (CKM)

As the population of CKD patients in Japan began increasing and aging, debate has arisen regarding who should be started on dialysis, and it has been recognized on a national level that some patients will ultimately forgo or withdraw from dialysis (or RRT). In 2020, this issue was brought into the spotlight through media coverage of a patient who died after withdrawal of maintenance dialysis, which prompted discussion of when it is appropriate to suspend medical treatment and what process should be followed. The case in question was exceptional because the patient was relatively young and was not in poor physical condition due to an illness like advanced cancer. Consequently, questions were raised as to whether the patient had been informed in advance that RRT was an option, and whether it would be ethical to allow someone not in a terminal condition to intentionally forgo dialysis.

In response to these developments, the JSDT published a “Proposal regarding the decision-making process for initiation and continuation of dialysis” in 2020. This document describes the selection of RRT for ESKD patients as well as the process for providing information about forgoing dialysis and making that decision [9]. When a patient needs some form of RRT, the decision should be made based on shared decision-making (SDM), and thorough advance care planning (ACP) involving repeated advance discussion of care plans with family members and the medical and supportive care team, with the goal of the medical team respecting the patient’s wishes throughout this process.

The proposal further states that patients who decline RRT should be provided information about conservative kidney management (CKM). CKM is a general term for not selecting RRT, which encompasses previously used expressions such as “forgoing dialysis,” “withdrawing from dialysis,” and “conservative treatment.” CKM is recognized as a treatment option for patients who are unlikely to benefit from dialysis (when dialysis cannot be performed safely and poses a high risk to the patient’s life) or who are in very poor general condition and have expressed their intent to forgo or withdraw from dialysis. However, the concept of CKM and how it is performed vary between countries [10]. In Japan, CKM refers to treatment and supportive care for patients who wish to forgo or withdraw from dialysis. Two clinical guides on CKM have been developed and published: a clinical guide entitled “Conservative Kidney Management for Elderly Patients with Renal Failure” and an official guide to “Palliative Care in Patients with Renal Failure” needed for patients withdrawn from dialysis.

## Japan Renal Replacement Therapy Association

The diversification of RRT and establishment of outpatient RRT options in clinical practice are urgent tasks that must be tackled through a multidisciplinary approach. In response to this situation, revisions to the payment system for medical services have increased the need for specialists to respond to trends in clinical practice and insurance-covered medical treatment, including reimbursements for starting RRT and for RRT counseling and management, which will help to promote peritoneal dialysis and renal transplantation. The Japan Renal Replacement Therapy Association (JRRTA) was established to promote the selection of appropriate RRT through multidisciplinary team medicine and to improve the activities of daily living (ADL) and quality of life (QOL) of dialysis patients and renal transplant recipients, with particular emphasis on promoting in-home options such as peritoneal dialysis as well as renal transplantation. For this reason, it was decided that the JRRTA would certify, train, and educate RRT Professional Instructors as medical professionals involved in dialysis therapy, with support and guidance not only from physicians but also other medical professionals involved in dialysis therapy, including nurses, clinical engineers, pharmacists, and dieticians.

The rapid changes in society overall and in the healthcare economics of dialysis therapy present us with new challenges and opportunities, and make the concept of SDGs relevant to our daily practice. The SDGs are the most fundamental part of the 2030 Agenda for Sustainable Development set by the United Nations. This Agenda, which was adopted by all united nations (UN) member states in 2015, provides “a shared blueprint for peace and prosperity for people and the planet, now and into the future” [11]. The SDGs, which consist of 17 items, are recognized by all UN member states and apply to everyone, for the benefit of all. To better meet the needs of patients, care providers must work in multidisciplinary teams and continue implementing tried-and-true approaches while simultaneously incorporating new concepts, techniques, and methodologies. The purpose of SDGs at the annual meeting is to create an overall picture of these needs and present it in a simple, accessible, memorable, and easy-to-implement framework. “What should be the key goals of the 67th Annual Meeting of the JSDT in Yokohama? What are the goals to be achieved? What is the timeline for achieving those goals?” Such important questions that needed to be resolved were raised, and answers to these questions were sought.

This special issue shares some of the papers presented at the conference under the theme “In Pursuit of SDGs for Dialysis Therapy.” In particular, it focuses on various circumstances and issues surrounding dialysis therapy as it is practiced today, including COVID-19, natural disasters, aging-related frailty, forgoing/withdrawing dialysis and SDM/ACP as an important part of that process, the roles of nurses and clinical engineers in supporting dialysis, artificial intelligence (AI) as a tool predicted to become essential for future advances, and the newly introduced RRT Professional Instructor certification system in Japan. We hope that the papers in this special issue will bring us much closer to finding the answers. As the guest editors, we would like to thank all the authors who contributed to this special issue.
